# Transmembrane domain interactions underlie NSG1 regulation of sortilin ectodomain shedding

**DOI:** 10.1016/j.jbc.2025.110804

**Published:** 2025-10-08

**Authors:** Malene Overby, Lasse Messell Desdorf, Lisbeth Kjølbye, Tommy Rosendahl, Jason Porter Weick, Birgit Schiøtt, Nils Anton Berglund, Heidi Kaastrup Müller

**Affiliations:** 1 Department of Clinical Medicine, Translational Neuropsychiatry Unit, Aarhus University, Aarhus, Denmark; 2 Department of Chemistry, Aarhus University, Aarhus, Denmark; 3 Department of Neurosciences, University of New Mexico School of Medicine, Albuquerque, New Mexico, USA; 4 Kvantify, Copenhagen, Denmark

**Keywords:** transmembrane domain interactions, protein–protein interaction, ectodomain shedding, proteolytic processing, sortilin, NSG1, Alzheimer’s disease

## Abstract

Sortilin is a single-pass transmembrane receptor involved in intracellular trafficking, neurotrophic signaling, and protein clearance pathways relevant to neurodegenerative disease. We recently identified the neuron-specific protein NSG1 as a selective modulator of sortilin function, promoting its ectodomain shedding *via* ADAM10. However, the molecular basis of this interaction remains unresolved. Here, we present a structural framework for NSG1-mediated regulation of sortilin shedding. Using mutagenesis, biochemical assays, and structural modeling, we mapped the interaction interface of NSG1 to the helical transmembrane domain (TMD) of sortilin. We show that NSG1 binds a specific interface within the sortilin TMD, modulating its susceptibility to ectodomain shedding. Mutational analysis revealed that substitutions in the central region of the sortilin TMD, particularly T770W and A773W, significantly reduce NSG1-dependent shedding without disrupting complex formation. Coarse-grained molecular dynamics simulations identified two potential binding interfaces on the sortilin TMD and demonstrated that the T770W mutation shifts the preferred interface, thereby diminishing the ability of NSG1 to promote proteolytic processing. Notably, the closely related protein NSG2 has a different preferred binding mode on the sortilin TMD and does not induce shedding, highlighting the functional specificity of NSG1. Our findings establish the TMD–TMD interaction as an important basis for NSG1-mediated regulation of sortilin shedding. This study advances our understanding of how transmembrane interactions govern substrate-specific a disintegrin and metalloproteinase proteolysis and provides new insight into the molecular control of sortilin function. Given the emerging role of sortilin in Alzheimer’s disease, these insights may help clarify how its processing is regulated in the diseased brain.

Single-pass transmembrane proteins play essential roles in cellular signaling and trafficking. While their extracellular domains mediate ligand recognition, emerging evidence highlights a functional importance of their transmembrane domains (TMDs) (1, 2). Beyond simply anchoring proteins in the membrane, TMDs can facilitate specific protein–protein interactions that influence receptor conformation, oligomerization, and downstream signaling. However, for many receptors, the mechanistic contribution of TMDs to their biological functions remains poorly understood.

Sortilin is a single-pass type I transmembrane protein belonging to the Vps10p domain receptor family. It is highly expressed in neurons and has critical roles in intracellular trafficking and neurotrophic signaling (3). Structurally, sortilin is composed of a large extracellular Vps10p domain for ligand binding, a short juxtamembrane region, a single-pass TMD, and a cytoplasmic tail (4). While its extracellular domain has been extensively studied, the precise functions of the sortilin TMD, particularly in mediating interactions with regulatory proteins, remain to be elucidated.

Sortilin is also emerging as a gene of interest in neurodegenerative diseases, with genetic studies linking *SORT1* variants to altered Alzheimer’s disease (AD) risk (5, 6), and functional evidence showing its role in regulating key AD-related processes, including the trafficking of β-secretase 1 and amyloid precursor protein (APP) (7, 8, 9), as well as facilitating amyloid-β clearance through binding to apolipoprotein E (10). Furthermore, sortilin forms complexes with p75NTR to mediate proapoptotic signaling (11, 12) and regulates progranulin uptake (13, 14), a pathway implicated in frontotemporal dementia (FTD). Sortilin undergoes proteolytic cleavage through a process known as ectodomain shedding, whereby a disintegrin and metalloproteinase (ADAM) family of metalloproteinases cleave the receptor near the extracellular juxtamembrane region (15, 16). Shedding modulates sortilin function and abundance at the plasma membrane, but the regulatory mechanisms controlling sortilin shedding are not fully understood.

We previously showed that two members of the neuron-specific gene (NSG) family, NSG1 and NSG2, physically interact with sortilin. Notably, NSG1, but not the closely related NSG2, selectively promotes ectodomain shedding of sortilin in an ADAM10-dependent manner (17). This specificity is particularly striking given that NSG1 also regulates APP cleavage *via* ADAM10/17 (18), suggesting a broader role for NSG1 in modulating the proteolytic processing of proteins implicated in AD. Intriguingly, cleavage fragments of both sortilin and APP accumulate in plaques in AD brains, with sortilin deposits increasing as the disease progresses (19, 20). These findings underscore the potential relevance of NSG1-mediated sortilin regulation in neurodegenerative pathogenesis.

Despite this functional insight, the molecular basis of the NSG1–sortilin interaction has been an unsolved issue.

In this study, we identified the TMD of sortilin as a functionally important interface for NSG1-mediated regulation of ADAM10-dependent ectodomain shedding. Through a combination of mutagenesis, cell-based shedding assays, and coarse-grained molecular dynamics (CG-MD) simulations, we systematically map the TMD interface and pinpoint residues critical for function. Specifically, we show that mutations in the central region of the sortilin TMD (T770W and A773W) diminish NSG1-dependent shedding without disrupting complex formation. Simulations reveal that these mutations shift NSG1’s preferred binding interface, thereby reducing its ability to promote sortilin cleavage.

Together, our findings uncover a previously unrecognized TMD-mediated regulatory mechanism by which NSG1 controls sortilin proteolysis. This study not only resolves the question about the mode of NSG1–sortilin interaction but also provides broader insight into how transmembrane contacts govern substrate selection and shedding by ADAM proteases.

## Results

### Generation of sortilin constructs for immunoprecipitation-based interaction studies

To investigate the biochemical nature of the interaction between sortilin and NSG1, we used a FLAG-tagged sortilin construct for protein purification. Tagging sortilin at the C terminus disrupts its correct localization and endocytosis, as Golgi-localized, gamma-adaptin ear-containing, ARF-binding proteins require a free C terminus to bind sortilin (21). Previous studies have shown that the propeptide (PRO) of sortilin prevents minor misfolding, and removing it impairs sortilin processing and transport within the secretory pathway (4, 22). We therefore decided to place the FLAG tag at the N terminus, after the signal peptide (SP) and PRO (between amino acids 77 and 78) and annotated this construct SP_PRO_FLAG_SORT1. This construct allows detection of the mature form of sortilin and its ectodomain without affecting total expression levels (Fig. S1).

**Figure 1:**
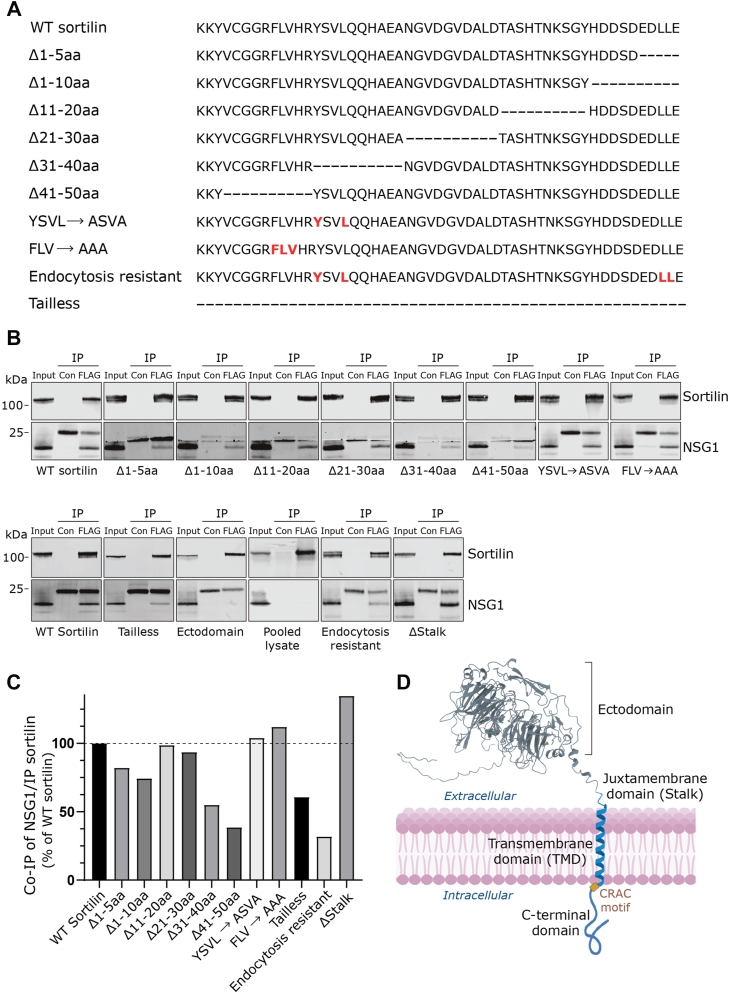
**Domain mapping identifies the TMD of sortilin as critical for NSG1 binding.***A*, schematic representation of the sortilin C-terminal deletion and mutation constructs. Construct annotations are shown on the *left*. Amino acids highlighted in *red* indicate substitutions with alanine, whereas *dashes* (-) denote deleted amino acids. *B*, coimmunoprecipitation (IP) of NSG1 with C-terminal–modified sortilin constructs. HEK293MSR cells overexpressing combinations of the indicated FLAG-tagged sortilin constructs with NSG1 were subjected to control immunoglobulin G and anti-FLAG antibody IP, to selectively pull-down FLAG-tagged sortilin. The precipitated immunocomplexes and corresponding input samples were subjected to Western blot analysis using the antibodies specified on the *right* of the blots. *C*, the amount of NSG1 coimmunoprecipitated with sortilin was quantified and normalized to the level of immunoprecipitated sortilin for each construct. Values are expressed as a percentage relative to the immunocomplex ratio for WT sortilin. *D*, structural representation of sortilin, highlighting its major domains: the extracellular domain, comprising the N-terminal ectodomain and the juxtamembrane domain (stalk), the TMD, CRAC motif, and the cytoplasmic C-terminal domain. CRAC, cholesterol recognition amino acid consensus; HEK, human embryonic kidney cell line; NSG1, neuron-specific gene 1; TMD, transmembrane domain.

We previously identified NSG1 as an interaction partner of sortilin using a yeast two-hybrid system optimized for detecting membrane-bound proteins and their interaction partners at the plasma membrane (17). The cytoplasmic C-terminal domain of sortilin (779–831 amino acids) was used as bait in this assay, leading us to hypothesize that the primary interaction site for NSG1 binding resides within this C-terminal region of sortilin. To test this hypothesis, we generated 10 different sortilin mutants containing small deletions (5–10 amino acids) or by substituting specific amino acids with alanine in endocytic sorting motifs (Fig. 1*A*). The dileucine (LL) motif and the C-terminal free glutamic acid (E) were deleted in only 3 of the 10 constructs, as these amino acids are important for binding GGA1–3 and proper protein trafficking (23). Specifically, two constructs with replacement mutations in sortilin endocytosis signals were included: YSVL→ASVA and FLV→AAA. These alterations target binding sites for adaptor protein complex 1 and 2 (24), the retromer complex, and group A P21-activated protein kinases (21). These trafficking-altered constructs served as controls to determine if changes in trafficking affected complex formation between sortilin and NSG1.

### Sortilin–NSG1 binding is driven by TMD interactions

To identify the domains responsible for the interaction with NSG1, we used immunoprecipitation to screen the 10 C-terminally altered sortilin constructs for complex formation with NSG1. The sortilin constructs were coexpressed with NSG1 in transiently transfected human embryonic kidney (HEK) 293MSR cells, and extracts were prepared using 5 mM CHAPS detergent. NSG1 was coimmunoprecipitated from the extracts with an anti-FLAG antibody recognizing the sortilin constructs, whereas an isotype control antibody did not coprecipitate NSG1 (Fig. 1, *B* and *C*). The results showed varying degrees of NSG1 precipitation among the C-terminally altered sortilin constructs compared with WT sortilin (Fig. 1, *B* and *C*). Specifically, the Δ1–5aa and Δ1–10aa constructs, which lack the extreme C-terminal part of sortilin, displayed reduced NSG1 binding, which may be due to the loss of the LL motif and the free glutamic acid (E). However, the Δ31–40aa and Δ41–50aa constructs exhibited even greater reductions in NSG1 precipitation. These two constructs each involved the deletion of YSVL and/or FVL, respectively, which are endocytic sorting motifs (Fig. 1*A*). To ensure that the diminished complex formation observed with these two constructs was not because of mislocalization of sortilin within cellular organelles, we used the YSVL->ASVA and FVL->AAA constructs as controls. These controls showed a similar binding of NSG1 as WT sortilin. Thus, they helped confirm that the reduced binding of NSG1 to the Δ31–40aa and Δ41–50aa constructs was specifically related to structural alterations and not to potential mislocalization effects.

In contrast, when mutations in the YSVL motif were combined with alanine substitutions in the C-terminal LL motif to create an endocytosis-resistant construct (YSVL→ASVA, LL→AA) (21), we observed the lowest NSG1 binding among all the constructs tested (Fig. 1, *B* and *C*). This suggests that impaired sortilin endocytosis reduces its association with NSG1. To our surprise, a sortilin tailless construct, lacking the entire cytoplasmic C-terminal domain, retained the ability to bind NSG1 (Fig. 1, *B* and *C*). Since NSG1 was originally identified as a binding partner for the C-terminal domain of sortilin (17), it suggests that the interaction between NSG1 and sortilin involves multiple binding interfaces.

Sortilin can be divided into four structural domains: the ectodomain, the juxtamembrane domain, the TMD, and the C-terminal domain (Fig. 1*D*). Since we previously demonstrated that NSG1 regulates ADAM10-dependent ectodomain shedding of sortilin, we tested a ΔStalk construct, where the extracellular juxtamembrane domain, also known as the cleavage site for ADAM10/17, was deleted (25, 26, 27). This construct formed complexes with NSG1 just as effectively as WT sortilin (Fig. 1, *B* and *C*). However, a construct consisting solely of the non–membrane-bound sortilin ectodomain did not form a complex with NSG1 (Fig. 1*B*). Together, these observations, along with the substantially reduced binding of NSG1 to the Δ31–40aa and Δ41–50aa constructs, which lack amino acid residues near the TMD, suggest that the TMD of sortilin plays an important role in its complex formation with NSG1. This conclusion was further supported by experiments in which we expressed NSG1 and sortilin in separate cells and then mixed the lysates before performing immunoprecipitation. No complex formation between NSG1 and sortilin was formed (Fig. 1*B*). This suggests that both proteins must be situated within the same membrane to form a complex, thus highlighting the involvement of the sortilin TMD in mediating NSG1 binding and linking the TMD of NSG1 as the initial interaction site with sortilin. It further demonstrates that protein dimerization depends not only on the TMD but also on the highly flexible regions adjacent to it.

### Mutations in the TMD of sortilin affect NSG1-mediated ectodomain shedding

**Figure 2:**
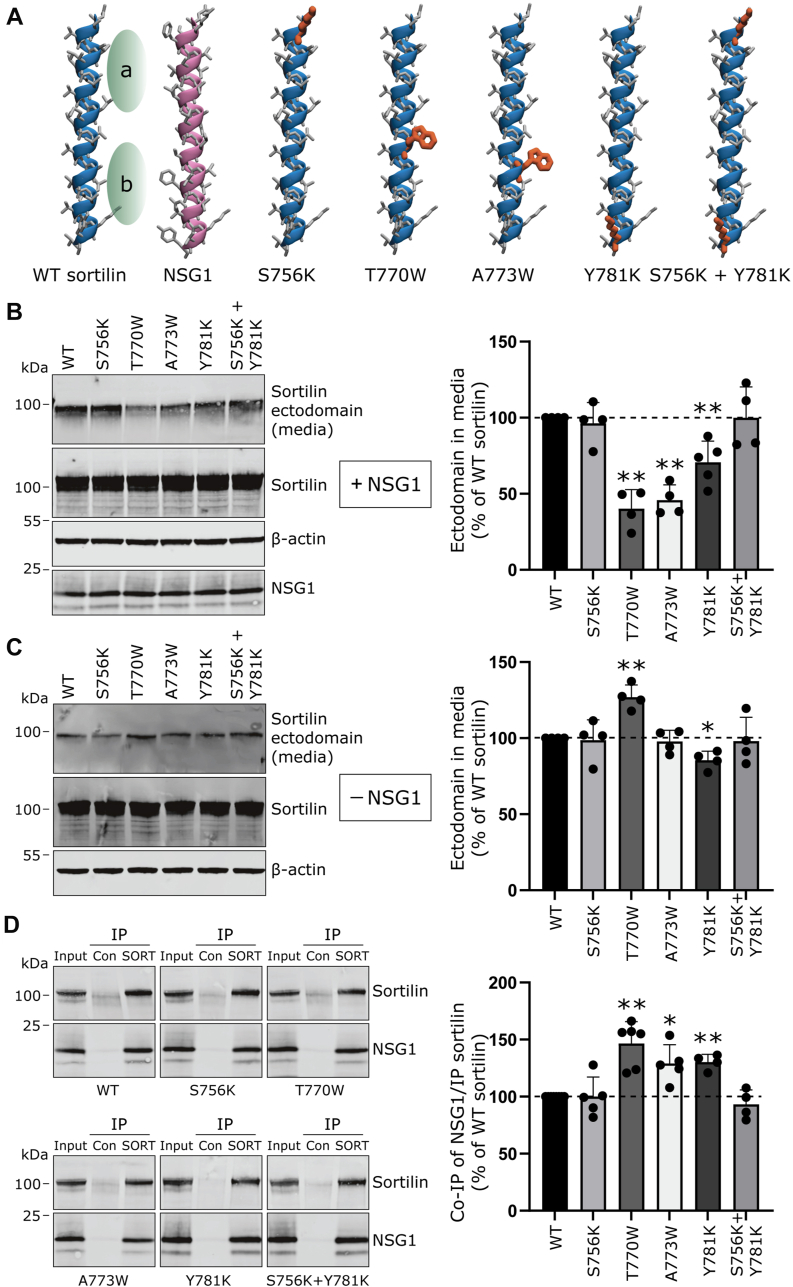
**Mutations in the TMD of sortilin differentially affect NSG1-mediated and basal ectodomain shedding.***A*, structural representation of the transmembrane helices of sortilin (*blue*) and NSG1 (*purple*). Side chains are shown as *gray sticks*. *Green areas*, labeled “a” and “b,” represent cavities within the sortilin TMD. NSG1 is positioned with its bulky residues oriented toward these sortilin cavities. Mutated residues in sortilin are highlighted in *orange*. *B*, *left*, representative Western blots of whole-cell lysates and conditioned media from HEK293MSR cells overexpressing sortilin with the indicated TMD mutations, along with NSG1. Antibodies used are specified on the *right*. *Right*, bar graph shows the relative abundance of sortilin ectodomain in the media, normalized to total sortilin, and expressed as a percentage of WT sortilin. Quantified results are from four independent experiments; one-sample *t* test, ∗∗*p* < 0.01 compared with WT sortilin. *C*, *left*, representative Western blots of whole-cell lysates and conditioned media from HEK293MSR cells overexpressing sortilin with the indicated TMD mutations (*without* NSG1). Antibodies used are indicated on the *right*. *Right*, bar graph showing the relative abundance of sortilin ectodomain in the media, normalized to total sortilin, and expressed as a percentage of WT sortilin. Quantified results are from four independent experiments; one-sample *t* test, ∗*p* < 0.05, ∗∗*p* < 0.01 compared with WT sortilin. *D*, *left*, coimmunoprecipitation (IP) of NSG1 with sortilin TMD mutants. HEK293MSR cells overexpressing combinations of sortilin with the indicated TMD mutations, along with NSG1, were subjected to control immunoglobulin G and anti-sortilin (SORT) antibody IP. The precipitated immunocomplexes and corresponding input samples were subjected to Western blot analysis using the antibodies specified on the *right* of the blots. *Right*, bar graph showing the amount of NSG1 coimmunoprecipitated with sortilin, normalized to the level of immunoprecipitated sortilin for each construct. Values are expressed as a percentage relative to the immunocomplex ratio for WT sortilin. Quantified results are from five independent experiments; one-sample *t* test, ∗*p* < 0.05, ∗∗*p* < 0.01 compared with WT sortilin. HEK, human embryonic kidney cell line; NSG1, neuron-specific gene 1; TMD, transmembrane domain.

To better understand the regulatory role of the TMD–TMD interaction between sortilin and NSG1, we aimed to identify specific amino acid residues mediating the assembly of their TMDs. To this end, we generated five constructs with targeted mutations in the TMD of WT sortilin that could potentially impact NSG1 binding (Fig. 2*A*). Since experimentally resolved structures for the TMDs of both sortilin and NSG1 are unavailable, we employed a *de novo* modeling approach to build molecular structures for each protein. The TMDs were modeled as straight right-handed α-helices (Fig. 2*A*) based on secondary structure predictions from the PROTEUS2 prediction tool (28) and supported by NMR data of single-pass transmembrane proteins, which commonly exhibit this secondary structure (29). The length of the TMDs was based on NMR studies showing that single-pass TMDs typically consist of 24 to 35 residues, sufficient to span the ∼4 nm (∼40 Å) hydrophobic thickness of a neuronal plasma membrane model (30). Our structural predictions were further validated against AlphaFold2 models (31), which aligned closely with our defined TMD (Fig. S2).

Upon examining the molecular structures of the sortilin and NSG1 TMDs, we hypothesized that their assembly is primarily driven by hydrophobic interactions and potentially *via* zipper-like motifs, where small amino acid residues form cavities that larger amino acid residues fit into. To disrupt these hydrophobic interactions, we introduced single amino acid residue substitutions in sortilin with either bulky or positively charged residues (Fig. 2*A*). Specifically, we introduced the positively charged lysine residues at positions S756 or Y781, either independently or simultaneously, near the two respective termini of the sortilin TMD, intending to repel the TMDs from each other and disrupt hydrophobic interactions. In particular, the lysine residue at Y781 could repel the negatively charged glutamic acid residue at the TMD terminus of NSG1. In addition, we replaced the threonine residue at position 770, a polar amino acid residue located within the second cavity of the sortilin TMD, with a tryptophan residue (T770W), a large and bulky amino acid residue, to disrupt the hypothesized zipper-like motif (Fig. 2*A*). Similarly, the alanine residue at position 773, also located in the second cavity, was substituted with a tryptophan residue (A773W) to interrupt the zipper-like motif (Fig. 2*A*). To determine whether these TMD mutations affected NSG1-mediated sortilin ectodomain shedding, we used Western blot analysis to measure the levels of accumulated sortilin ectodomain in conditioned media from HEK293MSR cells cotransfected with NSG1, using an assay previously established (17). Accumulation of sortilin ectodomain in media was significantly reduced for the TMD mutants T770W, A773W, and Y781K to 40%, 46%, and 71% of WT sortilin levels, respectively (Fig. 2*B*). Among the three positively charged variants, S756K, Y781K, and the double mutant S756K + S781K, only the Y781K mutation reduced ectodomain shedding. However, this effect was less pronounced compared with the internal TMD mutants T770W and A773W, suggesting that amino acid residues near the TMD termini are not critical for the TMD–TMD assembly or that introducing positive charges in these positions has minimal impact on the interaction. Thus, introducing a large and bulky amino acid residue within the internal region of the sortilin TMD had the most pronounced effect on ectodomain shedding.

To test if the decreased ectodomain shedding observed for the sortilin mutants was specific to NSG1-mediated ectodomain shedding, HEK293MSR cells were transfected with the sortilin TMD mutant constructs alone (*i.e.*, in the absence of NSG1), and the levels of accumulated sortilin ectodomain in conditioned media were measured. The T770W mutation increased ectodomain shedding to 127%, whereas the Y781K mutation decreased ectodomain shedding to 85% of WT sortilin levels (Fig. 2*C*). The other mutations did not affect ectodomain shedding (Fig. 2*C*).

In summary, the positive charge introduced at Y781K reduces sortilin ectodomain shedding both in the presence and absence of NSG1, indicating a general NSG1-independent effect. In contrast, introducing a bulky tryptophan residue at position 770 decreases ectodomain shedding in the presence of NSG1 but increases sortilin ectodomain shedding in its absence (Fig. 2, *B* and *C*). This suggests that NSG1 modulates the shedding process through a distinct mechanism sensitive to structural changes introduced by the tryptophan. Last, the tryptophan substitution at A773 specifically impacts sortilin ectodomain shedding only in the presence of NSG1, highlighting the importance of this amino acid residue for NSG1-mediated ectodomain shedding.

To investigate whether the sortilin TMD mutants affected the binding of full-length NSG1, we employed an immunoprecipitation assay once again. The TMD mutations T770W, A773W, and Y781K, which led to a decrease in sortilin ectodomain shedding in the presence of NSG1, unexpectedly showed enhanced complex formation with NSG1, increasing by 146%, 129%, and 130%, respectively (Fig. 2*D*). This result was surprising, as we initially hypothesized that the reduction in NSG1-mediated ectodomain shedding would be linked to a weakened interaction between sortilin and NSG1.

### MD simulations reveal two binding interfaces and corresponding key residues on sortilin’s TMD

To better understand how the TMD mutations of sortilin affect the interaction between sortilin and NSG1, we used MD simulations to evaluate the placement and orientation of the TMDs and their key amino acid residues that could control the interaction. Simulations of NSG2 and sortilin were performed to explore the functional differences between NSG1 and NSG2 in regulating sortilin ectodomain shedding. While both proteins bind to sortilin, only NSG1 induces ectodomain shedding, whereas NSG2 does not (17). This distinction was utilized to investigate whether the two proteins interact with sortilin through different binding interfaces and to determine how these differences in binding may influence their functional roles. The TMD helices of sortilin, NSG1, and NSG2 were simulated at a CG resolution in a neuronal membrane model (30), with water and 0.15 M NaCl salt concentration, to imitate a realistic environment for studying the TMD–TMD interaction. All simulations showed binding between sortilin and NSG1 or NSG2.

**Figure 3:**
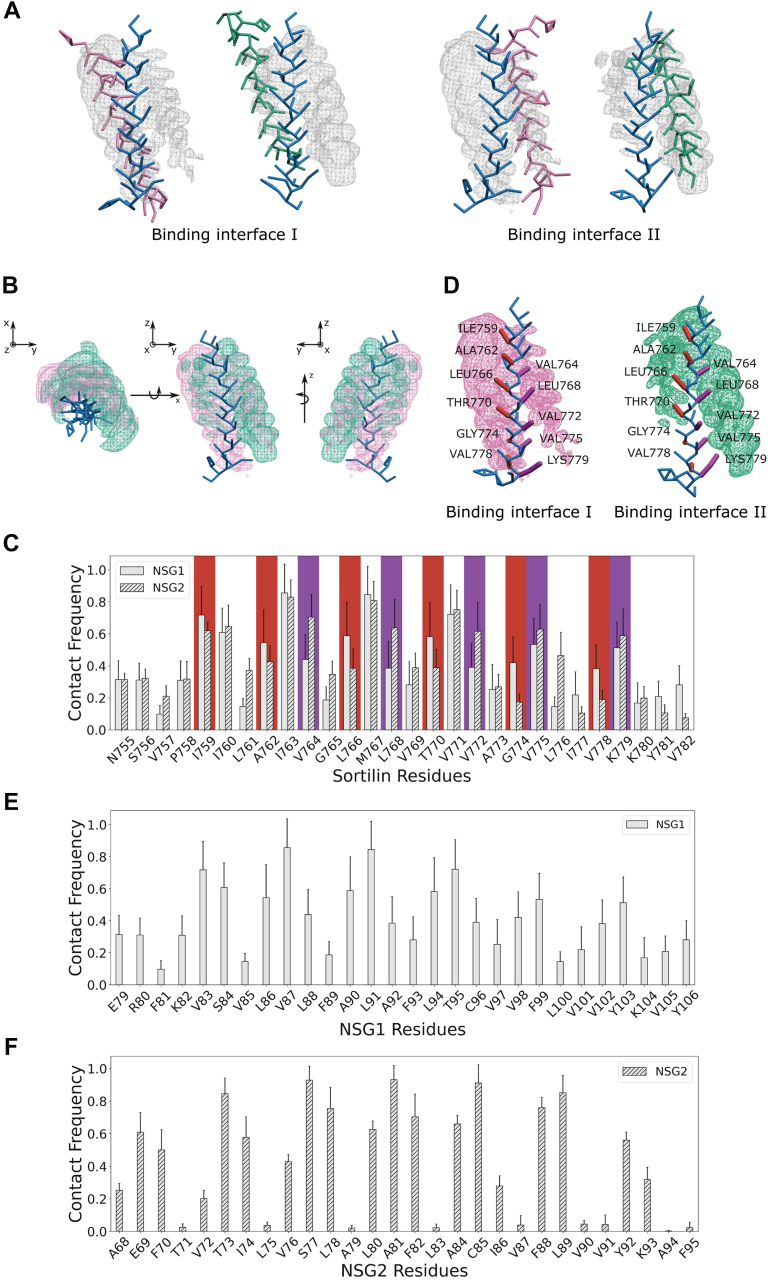
**NSG1 and NSG2 interact with distinct binding interfaces on the sortilin TMD, with differing occupancy levels.***A*, occupancy analysis of NSG1 (*purple*) and NSG2 (*green*) on the sortilin TMD. Mesh representation of occupancies, where the surface represents areas where NSG1 or NSG2 interacted with the sortilin TMD for more than 10% of the simulation time. On the *left*, NSG1 and NSG2 are positioned at binding interface I, and on the *right*, they are positioned at binding interface II, each visualized with its respective occupancy mesh. *B,* simultaneous visualization of NSG1 (*pink*) and NSG2 (*green*) occupancy maps, showing their overlap on the sortilin TMD. Occupancy maps are shown in the *xy* plane (*left*), the *yz* plane (*middle*), and the *yz* plane rotated 180° (*right*). *C*, contact frequency plots of NSG1 (*solid*) and NSG2 (*textured*) interactions with sortilin. Key residues in binding interface I are indicated by *red boxes*, and those in binding interface II are indicated with *purple boxes*. *D*, structural representation of binding interfaces I and II, showing key residues on the sortilin TMD. Amino acids essential for binding interface I are highlighted in *red*, whereas those for binding interface II are highlighted in *purple*. The occupancy mesh of NSG1 (*left*) and NSG2 (*right*) is visualized in *pink* and *green*, respectively. *E*, contact frequency plot of sortilin on NSG1. *F*, contact frequency plot of sortilin on NSG2. Contact frequency plots are based on 10 simulation repeats per system, and error bars represent SD. NSG1/2, neuron-specific gene 1/2; TMD, transmembrane domain.

Occupancy analysis of the MD simulations revealed two main binding interfaces on the TMD of sortilin, binding interfaces I and II (Fig. 3*A*). We found that both NSG1 and NSG2 can bind either interface; however, the results show a preferred binding interface depending on the binding partner. NSG1 prefers to occupy binding interface I to a greater extent than binding interface II, whereas the opposite was true for NSG2, which preferred binding interface II over I (Fig. 3*B*). The occupancy maps for NSG1 and NSG2 overlap, indicating the possibility that the TMDs may bind one interface and then transition to the other, depending on the binding partner.

The contact frequency analysis, based on a distance cutoff of 6 Å, further defined the binding interfaces between sortilin and NSG1 or NSG2 (Fig. 3*C*). The contact frequency plot shows that NSG1 and NSG2 have limited interactions at the termini of the TMD of sortilin (Fig. 3*C*). It also highlights the periodic pattern of the α-helix, namely that every third to fourth amino acid residue belongs to the same binding interface (Fig. 3*C*). Amino acid residues positioned between the two binding interfaces will display a high and similar contact frequency because the TMDs transition from one interface to the other and were therefore not seen as key amino acid residues for specificity in any of the binding interfaces, whereas amino acid residues specific to a binding interface will show a high and shifted contact frequency.

Binding interface I consists of amino acid residues I759, A762, L766, T770, G774, and V778 on sortilin; hence characterized mainly by hydrophobic amino acid residues besides one polar amino acid threonine (Fig. 3, *C* and *D*). Binding interface II consists of sortilin amino acid residues V764, L768, V772, V775, and K779, again a pattern of hydrophobic amino acid residues with one positively charged amino acid residue (Fig. 3, *C* and *D*).

The key amino acid residues on the TMD of NSG1 are V83, V87, L91, and T95, hence characterized by mainly hydrophobic amino acid residues besides one polar threonine residue, mostly located in the lower half of the TMD (Fig. 3*E*). The key amino acid residues on the TMD of NSG2 are E69, T73, S77, A81, C85, and L89, consequently characterized by hydrophobic and polar amino acid residues, a cysteine residue, and a negatively charged glutamic acid residue (Fig. 3*F*). The amino acid residues are equally distributed along the TMD (Fig. 3*F*), elucidating different molecular binding characteristics for the two TMD–TMD interactions with sortilin. In conclusion, NSG1 occupies binding interface I to a higher degree, with mainly hydrophobic interactions and polar interactions, whereas NSG2 occupies binding interface II to a higher degree, with hydrophobic and possible electrostatic interactions. It is notable that whilst the biophysical characteristics of interface I and interface II are highly similar and the sequence identity of the TMDs of NSG1 and NSG2 is over 70%, these subtle differences have a significant impact on their binding modes.

### The T770W mutation in the TMD of sortilin switches the NSG1 binding interface

**Figure 4:**
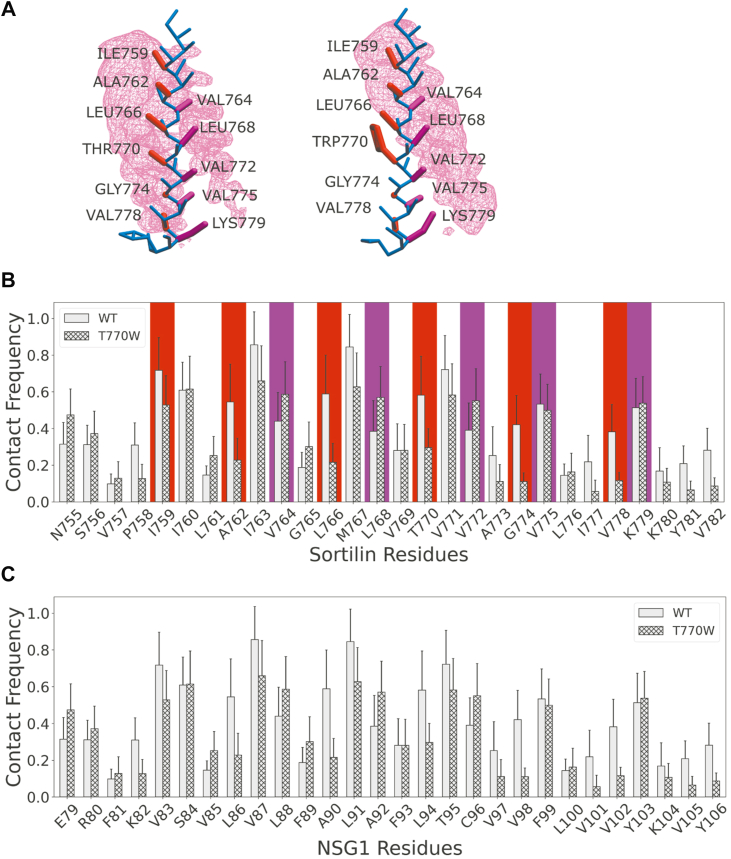
**T770W substitution in sortilin shifts NSG1 binding toward interface II.***A*, structural representation of NSG1 binding interfaces with WT sortilin (*left*) and sortilin containing the T770W mutation (*right*). Key residues in binding interface I are highlighted in *red*, whereas those at binding interface II are shown in *purple*. The *pink occupancy mesh* represents areas of NSG1 contact. *B*, contact frequency plots of NSG1 with WT sortilin (*solid*) and sortilin T770W mutant (*textured*). Key residues in binding interface I are indicated by *red boxes*, and those in binding interface II are indicated by *purple boxes*. *C*, contact frequency plots of WT sortilin (*solid*) and T770W (*textured*) on NSG1. Contact frequency plots are based on 10 simulation repeats per system, and error bars represent SD. NSG1, neuron-specific gene 1.

To better understand the biochemical nature of the interaction between NSG1 and the sortilin T770W mutant, we further analyzed this interaction through simulations. The T770W mutant was selected because of its strong reduction in ectodomain shedding but increased complex formation with NSG1, despite T770 being predicted as a key amino acid residue in binding interface I. Occupancy analysis from the MD simulations revealed that the bulky tryptophan at position 770 switches the preferred binding interface from interface I to exclusively interface II (Figs. 4*A*, S3). A contact frequency plot revealed that the key interacting amino acid residues of sortilin in this new configuration are the same as those defined for NSG2 when bound at interface II (Fig. 4*B*). In this altered interface, the NSG1 amino acid residues E79, S84, L88, A92, and C96 are involved in the interaction with sortilin (Fig. 4*C*), which mirrors the hydrophobic and polar characteristics, the cysteine and electrostatic interaction seen in NSG2 at binding interface II (Fig. 3*F*). This suggests that although the interaction between NSG1 and sortilin is maintained upon the T770W mutation, a specific protein interface is required to facilitate the full functional effect of NSG1.

## Discussion

The cell surface abundance of single-pass transmembrane proteins is tightly regulated by trafficking mechanisms and proteolytic cleavage. Dysregulation of these processes is linked to AD, where improper cleavage of APP by recognized regulatory proteins can lead to pathological accumulation of amyloid-β peptides (32). For sortilin, which is implicated in both AD and FTD, only a few regulatory proteins have been identified to date. All these bind to motifs within its C-terminal domain and modulate its surface expression through trafficking-dependent mechanisms. Our discovery of NSG1 was the first identification of a sortilin-interacting protein that specifically regulates its cell surface abundance, and consequently its function, through ectodomain shedding (17). In this study, we defined the TMDs of NSG1 and sortilin as essential contact sites associated with NSG1-dependent sortilin shedding. We utilized a series of sortilin constructs with mutations and deletions in combination with immunoprecipitation assays to systematically identify the regions required for NSG1 binding. We demonstrated that the ectodomain of sortilin did not possess intrinsic affinity for NSG1 on its own, ruling out the extracellular regions as the primary mediators of this interaction. This finding is critical, as it shifts the focus to the TMD and intracellular region. Notably, although NSG1 was originally identified as a binding partner of the cytoplasmic C-terminal region of sortilin (17), this domain proved dispensable for NSG1 binding. Thus, our results identify the TMD as a previously unrecognized interaction site for NSG1, while not excluding additional regulatory contributions from the cytosolic domain. This interpretation is consistent with the well-established principle that membrane proteins often engage through multiple interaction interfaces, including extracellular domains, cytosolic regions, and TMDs, with distinct contact sites contributing to different regulatory outcomes.

In addition, our experiments revealed that the proper subcellular localization of sortilin and NSG1 within the same membrane compartment is essential for their interaction. No interaction was observed when cell lysates from separately transfected cells were mixed and subjected to immunoprecipitation. This suggests that the proteins must co-reside in the same membrane to form a functional complex. This finding highlights the importance of spatial context in mediating TMD–TMD interactions, as membrane proximity likely enables the structural alignment and hydrophobic interactions necessary for complex formation.

Building on this information, we explored the characteristics driving the TMD–TMD interaction between sortilin and NSG1. TMD interactions are typically mediated by a combination of hydrophobic forces, hydrogen bonding, and specific sequence motifs such as GxxxG or other small amino acid residue patterns that facilitate close packing of helices (33, 34, 35, 36, 37). However, because the sortilin TMD sequence lacks the typical structural motifs commonly associated with TMD interactions, which could serve as obvious starting points for targeted mutations, we turned to computational modeling to predict specific amino acid residues within the sortilin TMD that might influence its assembly with NSG1.

Using *de novo* structural prediction tools, we examined the spatial distribution of amino acid residues in the two helices and identified potential interaction sites between them. Through this structural modeling, we identified several amino acid residues in the sortilin TMD that were predicted to contribute significantly to the stability of the interaction with NSG1. Among these, two internal amino acid residues in the sortilin TMD stood out: threonine at position 770 and alanine at position 773. Functional analyses of these amino acid residues through targeted mutations revealed a critical role of these amino acid residues in mediating the effect of NSG1 on sortilin ectodomain shedding. Threonine 770, a polar amino acid residue, has a hydroxyl group on its side chain that can form hydrogen bonds with nearby amino acid residues in the NSG1 TMD, though fully atomistic resolution simulations would be needed to confirm this. This interaction would likely provide a stabilizing force between the two helices, particularly within the hydrophobic membrane environment, where such polar interactions are less common and, therefore, impactful. In addition, threonine can participate in “zipper-like” motifs, where alternating hydrophobic and polar amino acid residues help stabilize helix–helix interactions. In this context, the polarity of threonine may act as an anchor point for stabilizing helix–helix interactions, contributing not only to the stability of the complex but also to its specificity. Alanine, on the other hand, has a small, nonpolar side chain, making it ideal for allowing alpha helices to pack closely together. The minimal steric hindrance of alanine facilitates a compact and stable interface between the helices. This role is underscored by the impairment in NSG1-mediated sortilin shedding observed when alanine is mutated to the bulkier tryptophan. Alanine residues are often conserved in transmembrane helical interfaces where maintaining a stable interaction is crucial for protein function (1, 38, 39). Indeed, we noted the conservation of this alanine throughout sortilin evolution, highlighting its importance for the structural integrity of sortilin and functional regulation by NSG1. This is in line with former studies showing that the conservation of TMDs of single-pass transmembrane proteins is higher in helix–helix interfaces (40). In contrast, substituting the tyrosine at position 781, located at the cytoplasmic border of the sortilin TMD, with a positively charged lysine residue reduced sortilin shedding independently of NSG1. This specific tyrosine residue is part of a cholesterol recognition amino acid consensus motif, defined by the sequence (L/V)-X_1−5_-(Y/F/W)-X_1−5_-(K/R) (41, 42). The cholesterol recognition amino acid consensus motif, situated within the cytoplasmic leaflet of the membrane bilayer (Fig. 1*D*), is primarily involved in cholesterol binding, a critical lipid component of cellular membranes (43, 44, 45). Therefore, mutating the central tyrosine to a lysine residue likely alters the structural stability of sortilin and membrane localization, potentially explaining its general effect on ectodomain shedding. Whether this overall effect is due to specific interactions of cholesterol with the proteins or merely the effect of cholesterol on the physical properties of the cell membrane is not yet clear. These findings provided crucial insights into the molecular basis of the sortilin and NSG1 TMD interaction, allowing us to identify functionally important amino acid residues.

To better understand the interactions, we used MD to simulate the TMD–TMD interaction between sortilin and NSG1 and NSG2, allowing us to identify two distinct binding interfaces on the TMD of sortilin. The MD simulations confirmed the importance of hydrophobic amino acid residues and their role in stabilizing the TMD–TMD interactions and that a "zipper-like" motif drives the TMD–TMD interactions in both binding interfaces, but with differing specificity. It is noteworthy that among the key amino acid residues that define the specificity of binding interface I is a glycine residue, which is known to predominantly occur in helix–helix interfaces (33, 46), and the threonine residue at position 770, which was predicted to be of significant value in the computational modeling. MD simulations revealed that NSG1 preferentially binds interface I, whereas NSG2 preferentially binds interface II. The positively charged lysine residue contributes to the specificity of binding interface II since it interacts with the negatively charged glutamic acid residue at position 69 on NSG2. Thus, by comparing the binding modes of NSG1 and NSG2, we highlight a potential structural and molecular explanation for their different effects on sortilin ectodomain shedding. Still, whether NSG1 possesses additional structural features beyond the TMD that promote shedding remains an open question.

The primary focus has been on the TMDs as an important interaction point. However, the proteins' extracellular and intracellular parts also hold the potential to play a role in the interaction affecting the TMD–TMD interface and, hence, shedding. To address whether the TMD–TMD interface is affected, full-length structures from AlphaFold (31) of sortilin, NSG1, and NSG2 were simulated, which confirmed similar interfaces identified in the TMD–TMD simulations (Figs. S4-S6).

It has become evident that the TMD of single-pass transmembrane proteins, like sortilin, is important in modulating their activity and function. Specifically, TM helices contain more than one distinct interface, which can enhance an active or inactive complex formation, which has been observed for integrins (47) and receptor tyrosine kinases (48, 49). For the integrins, it has even been shown that different binding interfaces can bind different interaction partners simultaneously and form complexes with distinct functions (47). It is, therefore, intriguing that sortilin has distinct interfaces for NSG1 and NSG2 since NSG1 induces ectodomain shedding, whereas NSG2 does not. This raises the possibility that NSG1 and NSG2, depending on their binding, may form distinct functional complexes with sortilin. Whether all three proteins can assemble into a single active complex, or whether NSG1 and NSG2 bind sortilin independently to modulate its activity or specificity, remains an intriguing question for future investigation.

To expand on our functional analyses of the T770W mutant, which revealed a critical role in mediating the effect of NSG1 on sortilin ectodomain shedding, without decreasing complex formation between sortilin and NSG1, MD simulations were used to understand the biochemical changes that the mutation facilitates. The MD simulations not only revealed a switch in the preferred binding interface of NSG1 from I to II but also a slight rotation of the NSG1 helix when binding to interface II. The negatively charged amino acid residue E79 interacts to a higher degree, whereas the positively charged amino acid residue K82 interacts to a lower degree with the helix of sortilin. It also shows the importance of K779 in contributing to the specificity of binding interface II. The switch in the preferred binding interface explains the immunoprecipitation results of the TMD mutations, showing that NSG1 binding persists but that a specific protein interface is required to facilitate its functional effect.

The T770W mutation underscores the importance of the TMD in NSG1-mediated sortilin ectodomain shedding and how a single TMD mutation can alter the helix–helix interaction and the regulatory mechanism of NSG1. There are several documented examples of TMD mutations that either activate or inactivate receptors by changing their binding interface (50, 51, 52, 53, 54) and have also proven important within ectodomain shedding. The TMD-driven interaction between ADAM17 and rhomboid 2 regulates ADAM17-dependent protein ectodomain shedding (it is the Rhbdf2^sin^ point mutation in the first TMD of rhomboid 2 that leads to loss of ADAM17 activity) but also TMD mutations of ADAM17 lead to inhibition of ectodomain shedding in a substrate-selective manner (55). This highlights the potential of targeting helix–helix interaction for substrate-specific modulation of ectodomain shedding by ADAM proteases. Understanding TMD interactions has been an attractive avenue for rational drug design (56, 57), and TMD-targeting peptides are being developed but are not yet approved by the Food and Drug Administration (57). The helix–helix interaction between NSG1 and sortilin reveals a previously unrecognized mechanism for modulating ADAM10-dependent ectodomain shedding of sortilin. This is particularly relevant given that sortilin is both a genetic risk factor for AD (6) and plaque-forming protein (19, 20). Therefore, modulating sortilin shedding *via* NSG1 could offer novel strategies for the treatment of neurodegenerative disorders.

In summary, our data identify the TMD of sortilin as a critical determinant for NSG1-mediated regulation of ADAM10-dependent ectodomain shedding. Mutations within the TMD of sortilin can alter this regulatory function by shifting NSG1’s preferred binding interface, highlighting the importance of helix–helix interactions in controlling substrate-specific proteolysis. These findings suggest that not only the presence of the NSG1–sortilin interaction but also the precise orientation and interface of this TMD-mediated contact are key determinants of shedding activity. The distinct binding preferences of NSG1 and NSG2 highlight a novel regulatory axis of sortilin proteolysis, offering a potential target in AD and FTD that warrants further investigation.

## Experimental procedures

### Antibodies

Rabbit anti-sortilin (ANT-009), which recognizes the extracellular N-terminal domain, was purchased from Alomone Labs. Mouse anti-NSG1 (sc-390654) was purchased from Santa Cruz Biotechnology. Mouse anti-FLAG (F1804) was purchased from Sigma. Mouse immunoglobulin G (IgG) control (#5415) and rabbit IgG control (#2729) antibodies were purchased from Cell Signaling Technology. Mouse anti-β-actin (926-42212) and IRDye-conjugated secondary antibodies were obtained from LI-COR Biosciences. Antibody specificity for quantitative analyses was validated using lysates from mock- and construct-specific transfected HEK293MSR cells. The specificity of the sortilin ectodomain signal was further confirmed using both C-terminal– and N-terminal–directed antibodies, as previously described (17).

### Expression constructs

The full-length human *SORT1* construct was provided by the laboratory of Olav Michael Andersen (Aarhus University) and cloned into the pcDNA3.1 vector. Human *NSG1* was subcloned from the pPR3 yeast two-hybrid library vector into pcDNA3, as previously described (17). The sortilin mutant constructs: Δ1–5aa, Δ5–10aa, Δ11–20aa, Δ21–30aa, Δ31–40aa, Δ41–50aa, YSVL->ASVA, FLV->AAA, endocytosis mutant, tailless, ectodomain, and ΔStalk were generated by site-directed mutagenesis. Primers were designed using Takara Bio’s online tool (https://www.takarabio.com/learning-centers/cloning/primer-design-and-other-tools) and purchased from Merck. Constructs were assembled using the In-Fusion Cloning Kit according to the manufacturer’s instructions (Takara Bio). All constructs were verified by Sanger sequencing (Eurofins Genomics LightRun service).

### Cell culture and transfection

GripTite 293 HEK293 MSR cells (Invitrogen) were maintained as monolayer cultures in Dulbecco's Modified Eagle’s medium (Sigma), supplemented with 10% fetal bovine serum (Sigma), 0.1 mM nonessential amino acids (Gibco), 1% penicillin–streptomycin (Sigma), and 600 μg/ml Geneticin (Apollo Scientific). Cells were incubated at 37 °C in a humidified atmosphere containing 5% CO_2_. Transfections were performed on adherent cells using 2 μg of DNA and EcoTransfect reagent (OZ Biosciences), following the manufacturer’s protocol. The pcDNA3.1 vector was used in all experiments to ensure consistent DNA input across conditions. Cells were routinely screened for mycoplasma contamination using a quantitative PCR–based service (Eurofins Genomics).

### SDS-PAGE and Western blotting

All procedures were performed as described (58). Aliquots of cell lysate were mixed with SDS sample buffer (125 mM Tris–HCl, pH 6.8, 30% glycerol, 6% SDS, 0.03% bromophenol blue, and 125 mM DTT) and incubated at 65 °C for 20 min. Proteins were separated using NuPAGE 10% Bis–Tris gels (Invitrogen) with either the MOPS or MES buffer system (Invitrogen), followed by transfer to 0.2 μm nitrocellulose membranes using the Trans-Blot Turbo Transfer System (Bio-Rad). Membranes were blocked in Odyssey Blocking Buffer (LI-COR Biosciences) and incubated overnight at 4 °C with primary antibodies (details provided under individual experiments). After washing, membranes were incubated for 1 h at room temperature with appropriate IRDye-conjugated secondary antibodies (LI-COR Biosciences). Infrared signals were detected using the Odyssey CLx imaging system, and band intensities were quantified with Image Studio Software (version 5.0).

### Coimmunoprecipitation

Forty-eight hours post-transfection, HEK293MSR cells were washed with ice-cold wash buffer (50 mM Tris–HCl, pH 7.4, 150 mM NaCl) and lysed in ice-cold lysis buffer (50 mM Tris–HCl, pH 7.4, 150 mM NaCl, 5 mM CHAPS, and 1× cOmplete protease inhibitor cocktail) at 4 °C for 45 min. Lysates were cleared by centrifugation at 12,000*g* for 10 min to remove detergent-insoluble material. Equal volumes of total protein lysate were incubated with 2 μg of either mouse anti-FLAG, rabbit anti-sortilin, or respective control antibodies (mouse IgG or rabbit IgG) under constant rotation at 4 °C for 2 h. Immunocomplexes were captured by incubating the lysates with prewashed protein A-agarose beads (Santa Cruz Biotechnology, sc-2001) overnight at 4 °C. Beads were then washed twice with lysis buffer, and bound proteins were eluted by incubating in SDS sample buffer at 50 °C for 20 min.

Aliquots of cleared total lysate and immunoprecipitated samples were analyzed by SDS-PAGE followed by Western blotting. The primary antibodies used were rabbit anti-sortilin (1:500 dilution; Alomone Labs) and mouse anti-NSG1 (1:1000 dilution; Santa Cruz Biotechnology).

### Shedding assay

Five hours post-transfection, the culture medium was replaced with one-third of the original volume of fresh medium to concentrate secreted proteins. Forty-eight hours after transfection, conditioned medium was collected and centrifuged at 6000 rpm for 10 min at 4 °C to remove cell debris. Cells were lysed in ice-cold lysis buffer (50 mM Tris–HCl, pH 7.4, 150 mM NaCl, 5 mM CHAPS, and 1× cOmplete protease inhibitor cocktail) at 4 °C for 45 min, and cleared by centrifugation at 12,000*g* for 10 min at 4 °C. Both cleared medium and cell lysates were mixed with SDS sample buffer, sonicated for 3 s, and subjected to SDS-PAGE and Western blotting as described previously. The primary antibodies used were rabbit anti-sortilin (1:500 dilution; Alomone Labs), mouse anti-β-actin (1:2000 dilution; LI-COR Biosciences), and mouse anti-NSG1 (1:1000 dilution; Santa Cruz Biotechnology).

### System setup

#### Transmembrane helix modeling

The transmembrane α-helices of sortilin, NSG1, and NSG2 were built using the Python package PeptideBuilder (59) based on the FASTA sequences corresponding to UniProt entries Q99523, P42857, and Q9Y328, respectively (60). The sortilin T770W mutant was generated similarly, incorporating the single amino acid substitution directly into the FASTA sequence.

#### Full-length structure modeling

Full-length structures of sortilin, NSG1, and NSG2 were obtained from the AlphaFold2 Protein Structure Database (61) using the same UniProt identifiers. To exclude predicted unstructured regions (predicted local distance difference test <50), sortilin was truncated at residues Met1–Cys86 and Val782–Glu831; NSG1 at Met1–Ile66; and NSG2 at Met1–Lys66 and Ala142–His171. The full structure of sortilin was further subjected to tertiary restraints using a Gō-like model with default settings (62) based on an all-atom contact map generated from the rCSU server (63).

#### System design

For both the TMD and full-length systems, the proteins were separated by 7 nm and had the same relative orientation in the membrane for all repeats. The proteins were inserted into a membrane composed of 44% cholesterol and 56% 1-palmitoyl-2-oleoyl-*sn*-glycero-3-phosphocholine using INSANE (64). This was done to mimic the cholesterol content typical of neuronal membranes in healthy young individuals (30). The system dimensions were 20 × 20 × 15 nm^3^, solvated with water, and neutralized with 0.15 M NaCl. All models were converted to the Martini 3 CG force field using vermouth (65, 66), with the secondary structure defined by DSSP (67, 68).

### MD simulations

All systems were minimized using the steepest descent algorithm. MD simulations were carried out using GROMACS, version 2022.4 (https://zenodo.org/records/6103568) employing the Martini 3 force field (65). The systems were equilibrated in NVT ensemble for first 100 ps with a time step of 10 fs with position restraints of 1000 kJ/mol × nm^2^ on the proteins, then for 500 ps, and then for 1 ns in the NPT ensemble with a time step of 20 fs, but otherwise same settings. This was done to relax the membrane surrounding the proteins. The systems were then subjected to two additional rounds of equilibration in the NPT. The first equilibration was for 1 ns with a time step of 10 fs, and the second equilibration lasted for 3 ns with a time step of 20 fs. The temperature was 310 K, and the pressure was 1 bar. The Berendsen thermostat and barostat were used for the equilibration steps (69). The position restraints on the proteins were removed prior to the production run. The production run lasted for 20 μs with 10 repeats for every system at a time step of 20 fs. The v-rescale thermostat (70) and the Parinello–Rahman barostat (71) were used at a temperature of 310 K and 1 bar. The LINCS constraint algorithm was used throughout all simulations (72). The system size was 20 × 20 × 15 nm^3^, solvated with water, and neutralized with 0.15 M NaCl.

### Data analysis

All data are presented as mean ± SD. Independent experiments were defined as conducted at separate time points, typically weeks or months apart. Statistical analyses were performed using GraphPad Prism 10 (GraphPad Software, Inc). For comparisons involving multiple groups, a one-sample *t* test was used when evaluating mean values expressed as a percentage of control across independent experiments.

Occupancy maps were generated using volmap tool of VMD (73). The volmaps were calculated based on all 10 repeats of each system. Isosurfaces were visualized at a threshold of 10%, indicating that the selected protein occupied a given volume for at least 10% of the total trajectory. The Python package MDTraj was used to calculate the contact frequency by measuring the distances between the α-helices within a 0.6 nm cutoff. Results were averaged and normalized across all 10 repeats of each system. Error bars represent SD. Azimuth angles were calculated for the TMs to assess the variability between repeats and the relative orientation in the membrane (Figs. S7-S9). The azimuth angle represents a projection of a TM given orientation in the *xy*-plane (membrane plane). In addition, the distance between TMs was calculated to observe if their starting position and orientation biased the binding interfaces. All data and simulation files are publicly available at Zenodo: https://doi.org/10.5281/zenodo.14178557.

## Data availability

For the computational work, a Zenodo depository (10.5281/zenodo.14178557) is available that includes:

• Analysis
  ○ Occupancy maps files for all systems (.dx format)
  ○ Script to calculate occupancy maps (.tcl format)
  ○ Contact frequency data for all systems (.npy format)
  ○ Script to calculate contact frequency (.ipynb format)
• Simulations
  ○ Structure files for all systems (.gro format)
  ○ Simulation files for all systems (.tpr format)
  ○ Topology files for all systems (.top format)
  ○ Index files for all systems (.ndx format)
• Parameters

Parameter files for all systems (.itp format)

## Supporting information

This article contains supporting information.

## Conflict of interest

The authors declare that they have no conflicts of interest with the contents of this article.
