# Supplementary material for: Transmembrane domain interactions underlie NSG1 regulation of sortilin ectodomain shedding
Source: J Biol Chem. 2025 Oct 8;301(11):110804. doi: 10.1016/j.jbc.2025.110804 (PMC12603737; doi:10.1016/j.jbc.2025.110804)
Supplement: Supporting Information [file mmc1.docx]

**Supporting Information Figures for**

**Transmembrane domain interactions underlie NSG1 regulation of sortilin ectodomain shedding**

Malene Overby, Lasse Messell Desdorf, Lisbeth Kjølbye, Tommy Rosendahl, Jason Porter Weick, Birgit Schiøtt, Nils Anton Berglund, Heidi Kaastrup Müller

**This file includes**:

Supporting Figure 1 (Figure S1)

Supporting Figure 2 (Figure S2)

Supporting Figure 3 (Figure S3)

Supporting Figure 4 (Figure S4)

Supporting Figure 5 (Figure S5)

Supporting Figure 6 (Figure S6)

Supporting Figure 7 (Figure S7)

Supporting Figure 8 (Figure S8)

Supporting Figure 9 (Figure S9)

**
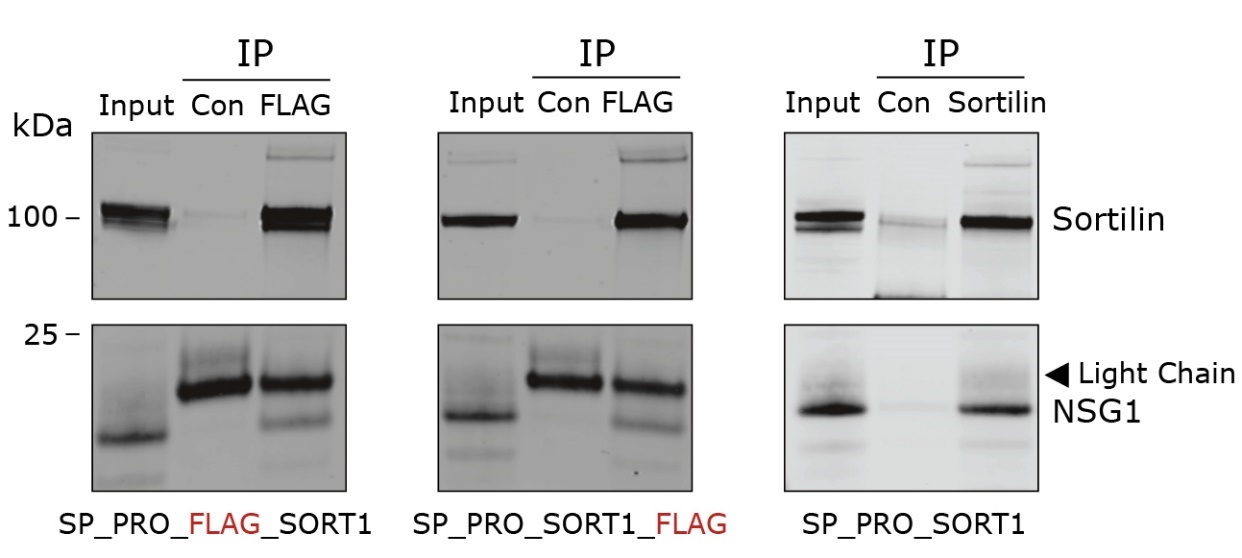
**

**Figure S1. Expression and immunoprecipitation of FLAG-tagged sortilin constructs**

HEK293MSR cells overexpressing NSG1 with sortilin constructs tagged with FLAG at the N-terminus (SP_PRO_FLAG_SORT1), C-terminus (SP_PRO_SORT1_FLAG), or without a tag (SP_PRO_SORT1) were subjected to immunoprecipitation (IP) with either mouse IgG control (Con) and anti-FLAG antibody (left and middle panels) or rabbit IgG control and anti-sortilin antibody (right panel). Immunocomplexes and corresponding input samples were analyzed by Western blot using antibodies against NSG1 and sortilin.

**
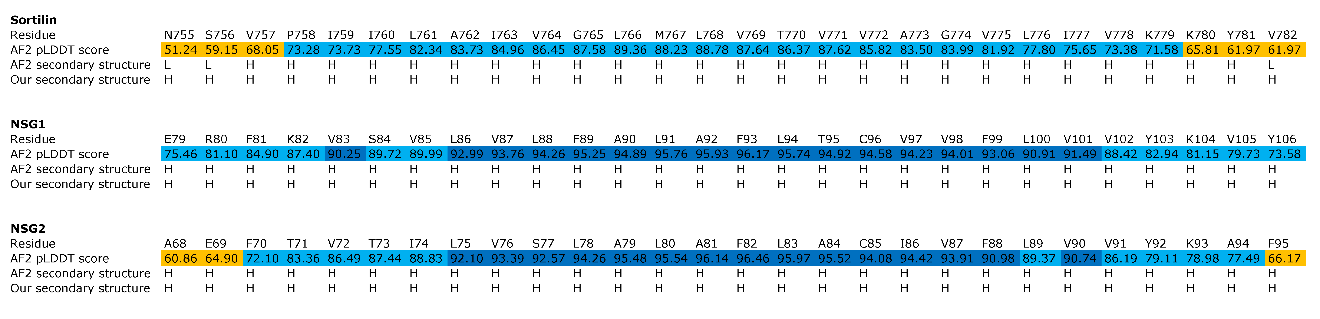
**

**Figure S2. Comparison of AlphaFold2 prediction of the TM domains and our de novo modeling**

The predicted secondary structure from the AlphaFold Database for sortilin, NSG1, and NSG2 alongside the pLDDT score of the structure compared with our de novo modeling of the secondary structure used for the MD simulations.


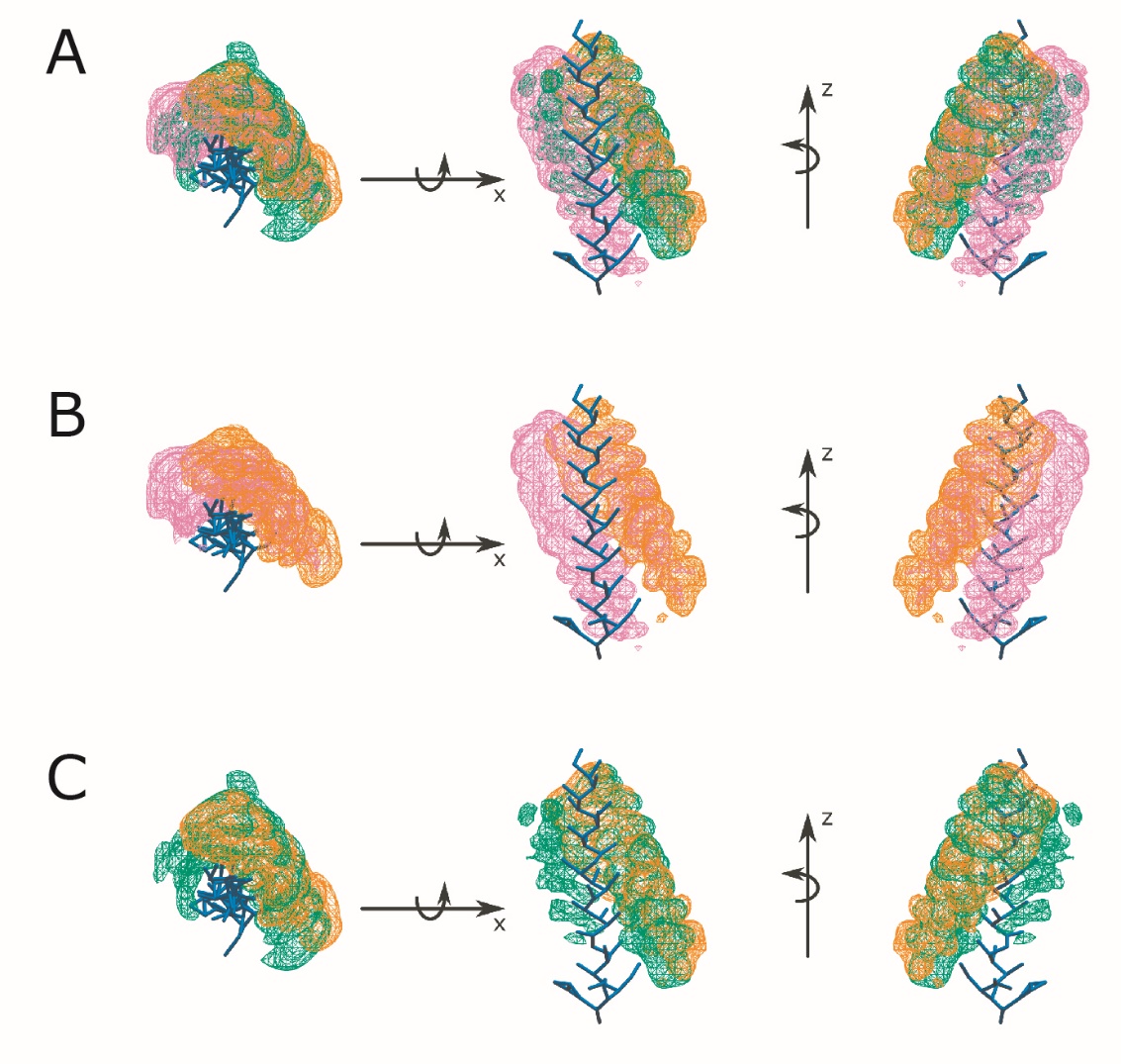


**Figure S3. Comparison of occupancy maps of NSG1 and NSG2 on WT sortilin and T770W mutant**

(A) Overlay of occupancy maps for NSG1 (pink) and NSG2 (green) on WT sortilin, along with NSG1 (orange) on sortilin T770W.

(B) Overlay of occupancy maps for NSG1 on WT sortilin (pink) and NSG1 on sortilin T770W (orange).

(C) Overlay of occupancy maps for NSG2 on WT sortilin (green) and NSG1 on sortilin T770W (orange).

For each comparison, occupancy maps are displayed in three orientations: the xy-plane (left), the yz-plane (middle), and the yz-plane rotated 180 degrees (right).


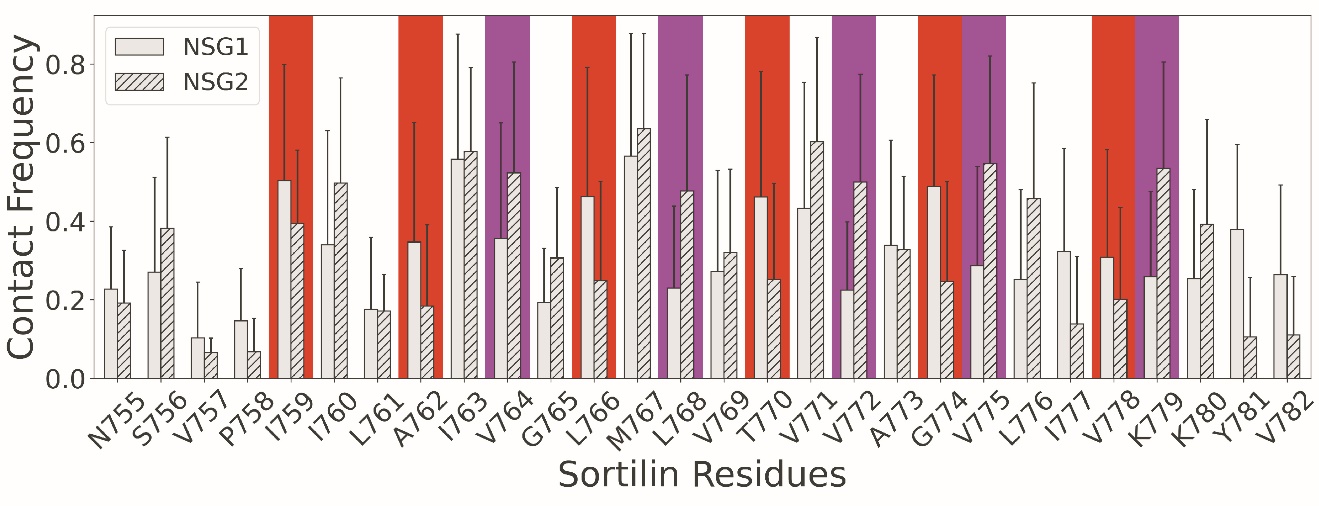


**Figure S4. Contact frequency plot for MD simulations of full-length sortilin with NSG1 and NSG2**

Contact frequency plots for MD simulations of full-length sortilin with NSG1 (solid) and NSG2 (textured) show trends similar to TMD simulations. Key amino acid residues in binding interface I are indicated with red boxes, and those in binding interface II with purple boxes. Each plot is based on ten simulations per system, with error bars representing SD.


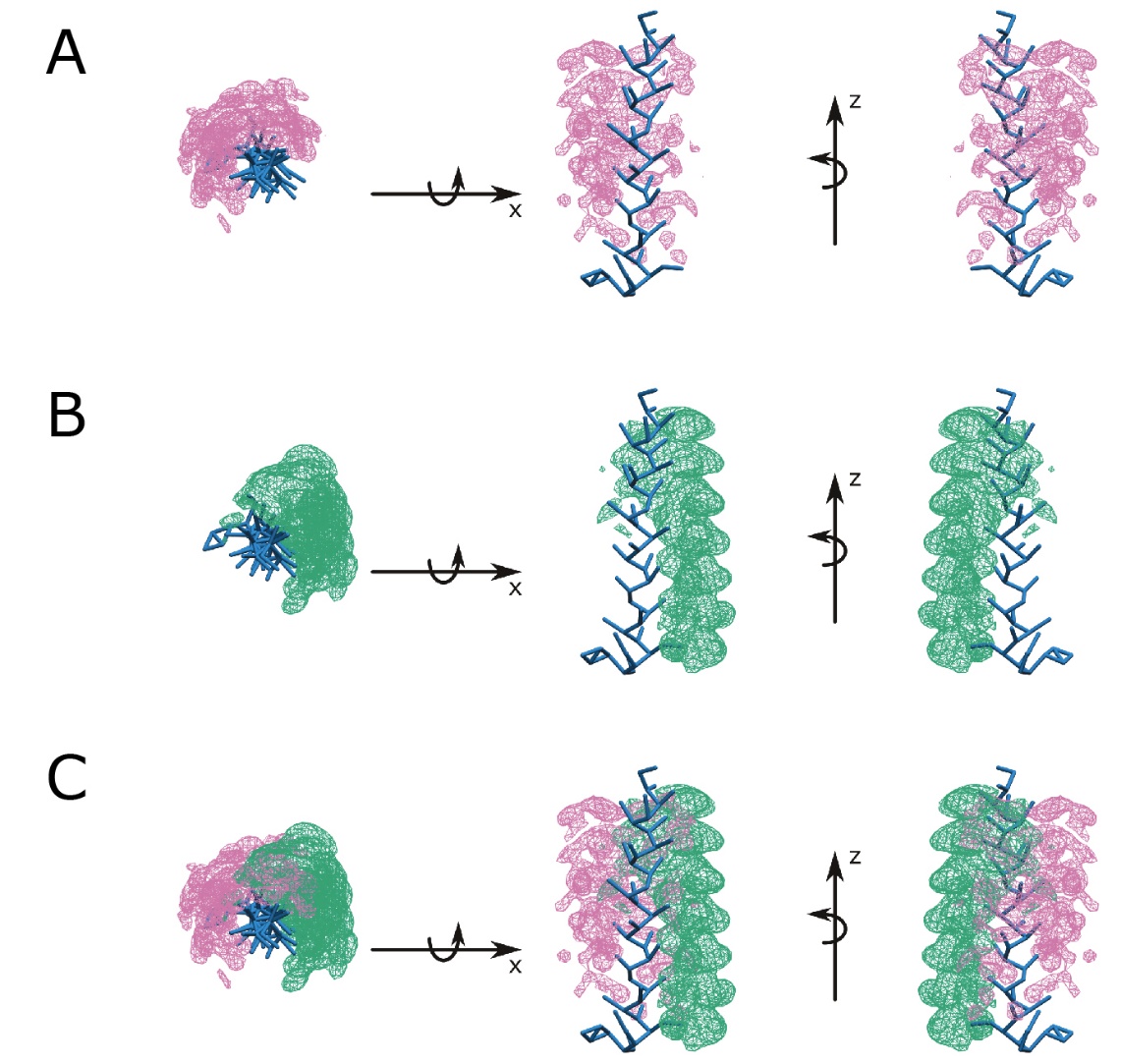


**Figure S5. Occupancy maps for full structure sortilin with NSG1 and NSG2**

(A) Occupancy mesh for NSG1 on sortilin is shown in pink.

(B) Occupancy mesh for NSG2 on sortilin is shown in green.

(C) Overlay of the occupancy mesh for NSG1 (pink) and NSG2 (green) on sortilin.

The occupancy maps are displayed in three orientations: the xy-plane (left), the yz-plane (middle), and the yz-plane rotated 180 degrees (right).


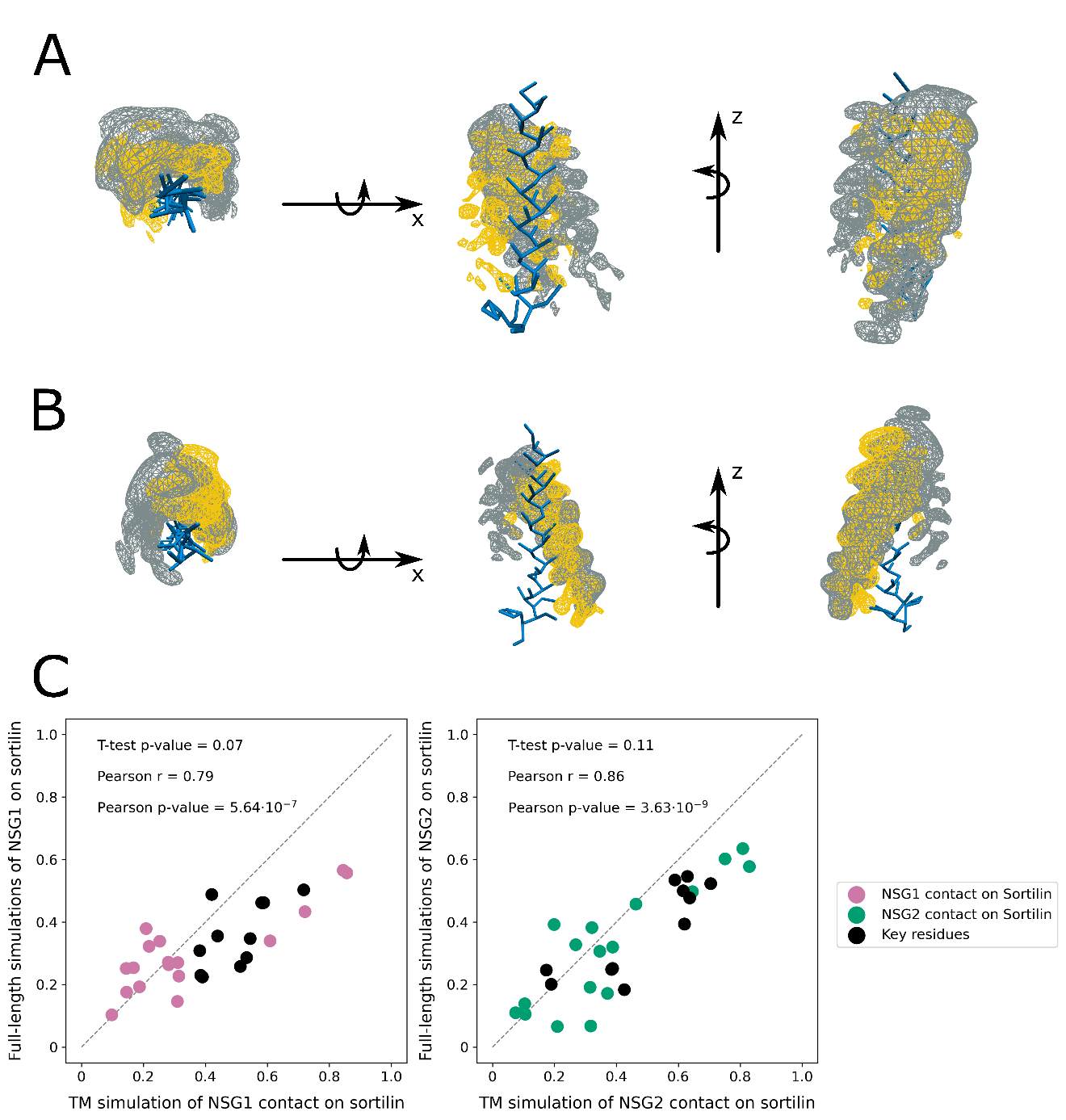


**Figure S6. Comparison of the TM simulations and full-length simulations**

(A) Occupancy mesh for NSG1 on sortilin. The occupancy mesh from the TM simulations is grey, while the full-length structures' occupancy mesh is yellow.

(B) Occupancy mesh for NSG2 on sortilin. The occupancy mesh from the TM simulations is grey, while the full-length structures' occupancy mesh is yellow.

(C) Contact frequency comparison of the two different simulation setups. The contact frequency of the TM simulations (x-axis) is plotted against the contact frequency of the full-length simulations (y-axis). The Shapiro-Wilk test was used to determine normality for the different samples, i.e. if the TM and FL data come from normal distributions. The statistic value for the NSG1 TM system was 0.95 with a p-value of 0.15, the NSG1 FL statistic value was 0.96 with a p-value of 0.35, the NSG2 TM statistic value was 0.95 with a p-value of 0.18, and the NSG2 FL statistic value was 0.95 with a p-value of 0.16. It was determined that the null hypothesis could not be rejected for the four samples. The Levene test was used to determine if the samples have equal variances, i.e. if the TM and FL data have equal variances. The statistic value for the NSG1 samples was 7.68 with a p-value of 0.01, and the statistic value for the NSG1 samples was 1.13 with a p-value of 0.29. It was determined that the null hypothesis must be rejected for the NSG1 samples, but not for the NSG2 samples. The Welch’s t-test was used on the NSG1 samples as they had unequal variances. The statistic value was 1.86 with a p-value of 0.07. The student t-test was used on the NSG2 samples as they had equal variances. The statistic value was 1.61 with a p-value of 0.11. In both cases the null hypothesis which says that the samples will have identical averages cannot be rejected. The Pearson test was used to test the linear correlation between TM and FL data. The NSG1 correlation coefficient was 0.79 with a p-value of 5.64 * 10^-7^. The NSG2 correlation coefficient was 0.86 with a p-value of 3.63 * 10^-9^. In both cases the null hypothesis which states that the samples are uncorrelated must be rejected. The dashed line indicates perfect agreement between the two simulation types. The statistical tests were calculated using the python package scipy [DOI: [10.1038/s41592-019-0686-2](https://doi.org/10.1038/s41592-019-0686-2)].


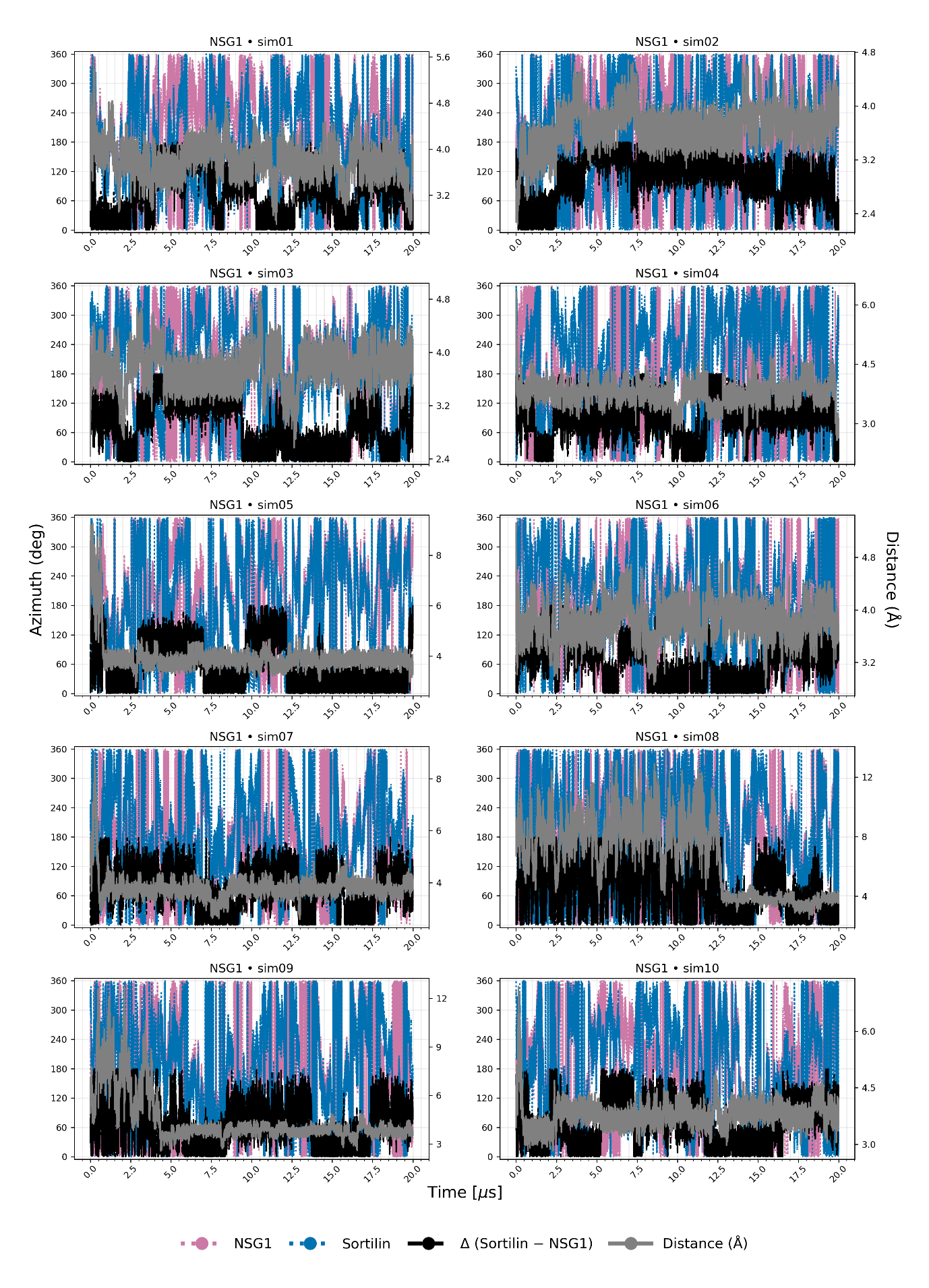


**Figure S7. NSG1 – Sortilin TM simulations**

Here the distance between sortilin and NSG1 (grey) is shown alongside their individual azimuth angle (pink and blue) and their subtracted azimuth angle (black). The distance represents the smallest distance between the two helices. The azimuth angle is calculated based on the middle residue, number 14, in the respective helices. This residue is made into a vector, which is projected into the xy-plane. The azimuth angle represents the ability of each helix to rotate around in the xy-plane. The subtracted azimuth angle represents the correlation between NSG1 and sortilin.


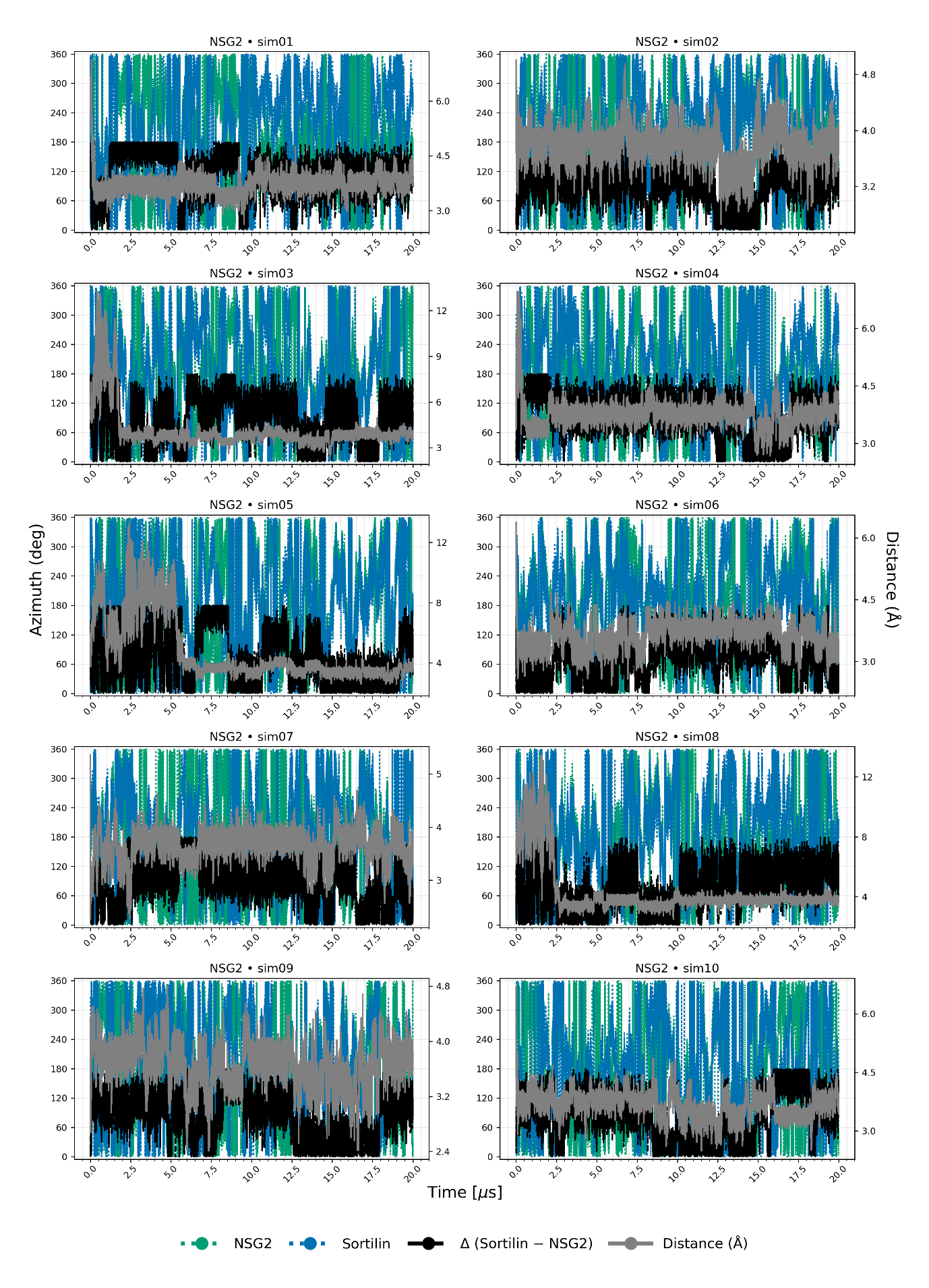


**Figure S8. NSG2 – Sortilin TM simulations**

Here the distance between sortilin and NSG2 (grey) is shown alongside their individual azimuth angle (green and blue) and their subtracted azimuth angle (black). The distance represents the smallest distance between the two helices. The azimuth angle is calculated based on the middle residue, number 14, in the respective helices. This residue is made into a vector, which is projected into the xy-plane. The azimuth angle represents the ability of each helix to rotate around in the xy-plane. The subtracted azimuth angle represents the correlation between NSG2 and sortilin.


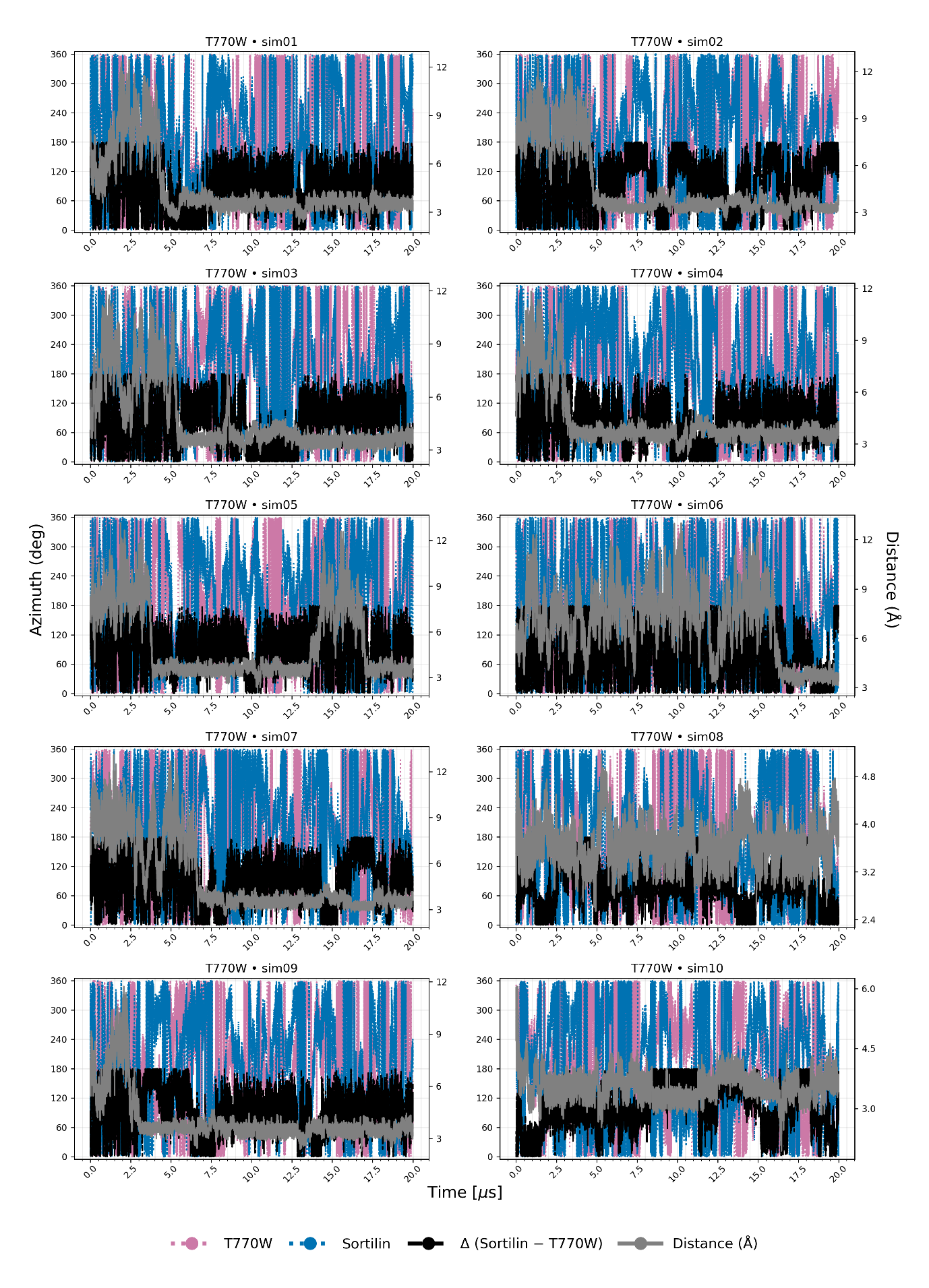


**Figure S9. NSG1 – mutated Sortilin TM simulations**

Here the distance between mutated sortilin and NSG1 (grey) is shown alongside their individual azimuth angle (pink and blue) and their subtracted azimuth angle (black). The distance represents the smallest distance between the two helices. The azimuth angle is calculated based on the middle residue, number 14, in the respective helices. This residue is made into a vector, which is projected into the xy-plane. The azimuth angle represents the ability of each helix to rotate around in the xy-plane. The subtracted azimuth angle represents the correlation between NSG1 and mutated sortilin.
